# Assessing the ability of large language models to simplify lumbar spine imaging reports into patient-facing text: a pilot study of GPT-4

**DOI:** 10.1007/s00256-025-05027-9

**Published:** 2025-09-09

**Authors:** Rushmin Khazanchi, Austin R. Chen, Parth Desai, Daniel Herrera, Jacob R. Staub, Matthew A. Follett, Mykhaylo Krushelnytskyy, Hanna Kemeny, Wellington K. Hsu, Alpesh A. Patel, Srikanth N. Divi

**Affiliations:** 1 Feinberg School of Medicine, Northwestern University, 676 N. St. Clair Street, Suite 1350, Chicago, IL 60611 USA; 2 Department of Orthopaedic Surgery, Northwestern University, Chicago, IL USA; 3 Department of Neurological Surgery, Northwestern University, Chicago, IL USA

**Keywords:** Artificial intelligence, Large language models, Radiology, GPT, Lumbar spine, MRI, Reading level

## Abstract

**Objective:**

To assess the ability of large language models (LLMs) to accurately simplify lumbar spine magnetic resonance imaging (MRI) reports.

**Materials and methods:**

Patients who underwent lumbar decompression and/or fusion surgery in 2022 at one tertiary academic medical center were queried using appropriate CPT codes. We then identified all patients with a preoperative ICD diagnosis of lumbar spondylolisthesis and extracted the latest preoperative spine MRI radiology report text. The GPT-4 API was deployed on deidentified reports with a prompt to produce translations and evaluated for accuracy and readability. An enhanced GPT prompt was constructed using high-scoring reports and evaluated on low-scoring reports.

**Results:**

Of 93 included reports, GPT effectively reduced the average reading level (11.47 versus 8.50, *p* < 0.001). While most reports had no accuracy issues, 34% of translations omitted at least one clinically relevant piece of information, while 6% produced a clinically significant inaccuracy in the translation. An enhanced prompt model using high scoring reports-maintained reading level while significantly improving omission rate (*p* < 0.0001). However, even in the enhanced prompt model, GPT made several errors regarding location of stenosis, description of prior spine surgery, and description of other spine pathologies.

**Conclusion:**

GPT-4 effectively simplifies the reading level of lumbar spine MRI reports. The model tends to omit key information in its translations, which can be mitigated with enhanced prompting. Further validation in the domain of spine radiology needs to be performed to facilitate clinical integration.

## Introduction

In the past decade, large language models (LLMs) like GPT-4 have rapidly advanced and garnered attention for their potential applications in healthcare. One of the critical areas of focus has been improving communication between healthcare providers and patients, especially in complex fields such as spine surgery, where imaging reports often guide crucial decisions [1, 2]. However, traditional imaging reports are written for medical professionals, leaving many patients unable to comprehend their meaning entirely. The burden of translating these complex medical findings into patient-friendly language can add significantly to a surgeon’s workload [3, 4]. With the increasing availability of electronic health data, timely, accurate, and understandable communication of health information has become an imperative goal [5].

Researchers have yet to determine how well LLMs can fully bridge this communication gap, especially when summarizing complex radiological findings like those in preoperative spine MRI reports. While some studies have demonstrated GPT-4’s superior ability to simplify radiology reports, issues such as omissions of essential findings and factual inconsistencies persist [6–8]. These limitations raise concerns about the safety and accuracy of relying on LLMs without proper regulatory oversight and human guidance to ensure ethical and practical implementation in clinical practice [1, 9]. Moreover, compared to radiologists, GPT-4 often fails to achieve the same level of accuracy in generating impressions, although its ability to simplify language has been well documented [10, 11].

Accordingly, this study aims to assess GPT-4’s ability to automate the creation of patient-facing summaries for preoperative spine MRI reports. By evaluating key parameters such as readability, word count, and the accuracy of simplified outputs, this research aims to determine whether GPT-4 can improve readability while maintaining clinical accuracy and completeness [4, 12].

## Materials and methods

### Data source

Patients undergoing lumbar decompression and/or fusion surgery in 2022 were queried retrospectively from our institutional database using the following procedural codes (CPT 63030, 63035, 63042, 63047, 63048, 22612, 22614, 22630, 22633, 22558, 22585) in addition to a diagnosis of lumbar spondylolisthesis (ICD-10 M43.16) among their active diagnoses at time of surgery. For the resulting set of patients, the latest preoperative spine MRI radiologist report prior to the date of surgery was extracted. Any patients where the MRI report could not be retrieved were excluded from analysis. The operative reports were fully de-identified prior to any additional analysis.

### GPT deployment

We passed the processed reports into the GPT-4 API (gpt-4–0613) with the following base prompt: “Please translate a radiology report into plain language that is easy to understand”. Default model settings were used, with the exception of temperature (a measure of output randomness) which was set to 0 to provide more deterministic results. A trained orthopaedic spine surgeon reviewed the resulting translations for accuracy of content. The presence of major inaccuracies, major omissions, minor inaccuracies, minor omissions, and an overall qualitative Likert score ranging from 1 to 5 were tabulated. A major inaccuracy or omission was described as incorrectly describing or failure to describe compression of nerve (at each level), anterolisthesis/facet joint effusion/signs of instability (each level), severity of stenosis (each level), location of stenosis (each level), existing spine instrumentation or prior spine surgery, and/or any other aspects of the initial radiology report that would impact clinical decision-making or shared decision-making. A minor inaccuracy or omission was defined as incorrectly describing or failure to describe presence of mild/moderate degenerative changes (not specific to level), presence of mild/moderate canal narrowing (not specific to level), patient-level explanation of spine pathology, any of the major criteria, though with some degree of information that may be clinically acceptable even if not fully complete, and/or any other aspects of the initial radiology report of academic or trivial importance.

After this initial review, we constructed an enhanced prompt using examples of high scoring reports (defined as reports with a Likert score of 5 as well as no omissions or inaccuracies) for the model to use as a template for its responses. This was done by randomly selecting a sample of five reports per model initialization to use as additional prompt context, and concatenating these examples to the original prompt The model was then redeployed on lower-scoring reports. Each of the resulting sets of report translations were again reviewed for accuracy along the same parameters as the initial review.

For each prompt iteration as well as for the original imaging report text, we conducted a readability analysis by tabulating the total word count as well as the Flesh-Kincaid reading level, a measure of text readability scaled by grades in the United States schooling system.

### Data analysis

The major and minor inaccuracy and omission rates, along with the Likert scores, were compared across all report iterations using McNemar Tests and Wilcoxon Ranked Sum tests, respectively. The word counts as well as the Flesh-Kincaid reading levels, were compared across all report iterations as well as with the original report text using paired two-tailed Welch’s t-tests. A Bonferroni correction for multiple hypothesis testing (*n* = 9) was applied, resulting in an adjusted threshold for significance of 0.006. Qualitative examples and observations of GPT failures were also assessed and categorized.

## Results

A total of 93 lumbar spine imaging radiology reports met our inclusion criteria. The original report text had an average word count of 412.45 words (SD: 172.59) with an average Flesch Kincaid reading level between 11 and 12th grade (11.47, SD 1.44) (Table 1). With the base prompt, the average word count was not significantly changed (399.64 SD 72.70, *p* = 0.510) though the average reading level was significantly decreased to between 8 and 9th grade (8.50 SD 1.27, *p* < 0.001). Of the 93 reports reviewed, 35% had a major omission, 20% had a minor omission, 6% had a major inaccuracy, and 3% had a minor inaccuracy. The average Likert score for the reports was a 3.48 (SD 1.56). Examples of these errors are provided in Table 2.

**Table 1:** GPT performance using base prompting strategy (*n* = 93)

	Original reports	Base prompt	*p*-value
Average word count (SD)	412.45 (172.59)	399.62 (72.70)	0.510
Average Flesch-Kincaid grade level (SD)	11.47 (1.44)	8.50 (1.27)	< 0.001*
Average Likert score (SD)	-	3.48 (1.56)	-
# of reports with minor omissions (%)	-	19 (20)	-
# of reports with major omissions (%)	-	33 (35)	-
# of reports with minor inaccuracies (%)	-	3 (3)	-
# of reports with major inaccuracies (%)	-	6 (6)	-

**Table 2:** Examples of GPT errors with base prompting strategy

Error type	Original report text versus GPT translation*
Major inaccuracy *GPT inaccurately describes prior spine surgery history and postsurgical changes*	**Actual:** *Postsurgical changes of double strut graft placement from T12 to L2 for a chronic fracture deformity of the L1 vertebral body and a left hemilaminectomy of L4* **GPT:** *The report concludes that there are post-surgical changes due to the graft placement and previous surgery at L4*
Major omission *GPT overgeneralizes and omits key localization information of spine pathology*	**Actual:** *At T12-L1, there is no significant central canal stenosis or neural foraminal narrowing. At L1-L2, there is a diffuse disc bulge with superimposed right foraminal disc protrusion with annular fissure as well as ligamentous hypertrophy and facet arthrosis without significant central canal stenosis. Spurring with mild to moderate right-sided neural foraminal narrowing. At L2-L3, there is ligamentous hypertrophy and facet arthrosis without significant central canal stenosis or neural foraminal narrowing. At L3-L4, there is a mild diffuse disc bulge asymmetric to the left with ligamentous hypertrophy and facet arthrosis without significant central canal stenosis. Mild lateral recess stenosis. Spurring with mild to moderate left-sided neural foraminal narrowing. At L4-L5, there is a diffuse disc bulge asymmetric left with prominent ligamentous hypertrophy and facet arthrosis causing moderate central canal and lateral recess stenosis, greater to the left. Spurring with moderate left-sided neural foraminal narrowing. At L5-S1, there is a mild diffuse disc bulge asymmetric to the right with ligamentous hypertrophy and facet arthrosis without significant central canal stenosis* **GPT:** *At the levels of the 1 st and 2nd, 2nd and 3rd, 3rd and 4th, 4th and 5th lumbar vertebrae, and 5th lumbar and 1 st sacral vertebrae, there are bulging discs, thickening of the ligaments, and arthritis of the joints, causing various degrees of narrowing of the spinal canal and the openings where the nerves exit the spine*
Minor inaccuracy *GPT makes an inaccurate statement in attempting to explain spine pathology*	**Actual:** *Fatty atrophy of the posterior paraspinal muscles* **GPT:** *The muscles at the back of the spine have lost some of their normal fat content*
Minor omission *GPT omits the fact that this radiographically appears to be a lesions*	**Actual:** *A 2.2 mm well-circumscribed lesion in the right inferior sacral ala is indeterminate and may represent an atypical hemangioma or other nonaggressive bony lesion in the absence of known malignancy. Consider short-term follow-up with contrast to ensure stability* **GPT:** *…a small area in the right lower part of the sacrum that's not clearly identified. It's recommended to do a follow-up scan with contrast to check this area*

^*^Translations derived from the base prompt analysis

Of these 93 reports, 36 were indicated to have had no errors. These 36 were used as template examples for GPT in the enhanced prompt. Within the remaining subgroup of 57 reports, the enhanced prompt returned a significantly higher word count (455.32 SD 77.35 versus 380.70 SD 65.16, *p* < 0.001) but a similar readability level (9.01 SD 1.27 versus 8.48 SD 1.34, *p* = 0.031) compared to the base prompt (Table 3). The average Likert score (4.39 SD 1.28 versus 2.47 SD 1.23, *p* < 0.001), major omission rate (7% versus 58%, *p* < 0.001) and minor omission rate (4% versus 33%, *p* < 0.001) were all improved relative to the base prompt. There was no significant change in the minor or major inaccuracy rate.

**Table 3:** GPT performance using enhanced prompting strategy (*n* = 57)

	Base prompt	Enhanced prompt	*p*-value
Average word count (SD)	380.70 (65.16)	455.32 (77.35)	< 0.001*
Average Flesch-Kincaid grade level (SD)	8.48 (1.34)	9.01 (1.27)	0.031
Average Likert score (SD)	2.47 (1.23)	4.39 (1.28)	< 0.001*
# of reports with minor omissions (%)	19 (33)	2 (4)	< 0.001*
# of reports with major omissions (%)	33 (58)	4 (7)	< 0.001*
# of reports with minor inaccuracies (%)	3 (5)	2 (4)	1.000
# of reports with major inaccuracies (%)	6 (11)	2 (4)	0.201

In analyzing the errors present in the enhanced prompt, three report translations failed to report the location of spinal stenosis (Table 4). An additional two translations failed to describe prior spine surgery, one translation failed to describe existing hardware in the spine, and one incorrectly described prior spine surgery. There were a series of reports that provided inaccurate descriptions of spine pathology including fatty atrophy, grade 2 anterolisthesis, and a potential abscess.

**Table 4:** Categorization of GPT errors with enhanced prompting strategy

Grouping	Type of error	Description	Count
Localization	Omission	Failure to describe level of spinal stenosis	3
Prior surgery	Omission and inaccuracy	Failure to describe prior spine surgery (2), existing spine instrumentation (1). Incorrect description of prior spine surgery (1)	4
Spine pathology	Inaccuracy	Incorrect description of fatty atrophy (1), lumbosacral anatomy (1), and a potential abscess (1)	3

## Discussion

The introduction of the 21 st Century Cures Act has provided patients with ready access to their health information, including radiology reports. Over time, this has translated into patients spending more time asking about specific details in their report during their encounters with their surgeon. However, radiology reports are intended as communications between medical professionals and therefore incorporate complex terminology and are written at a higher reading level. One of the major tasks a surgeon faces is to distill patient imaging and interpret relevant findings in the context of patient symptoms and physical exam. Given the ever-increasing time constraints faced by surgical practices, translating radiology reports for a patient can significantly add to a surgeon's workload. Therefore, the availability of a clinical tool to simplify terminology and improve readability and understanding of imaging reports for patients can be valuable. The benefits of this automated simplification must be balanced against the risk of “hallucinations” and providing patients with inaccurate medical information. Early stages of LLM integration into radiology will likely require significant radiologist oversight.

This study applies GPT-4 to lumbar spine MRI imaging reports to simplify radiologist text into patient-facing text. Automating this process effectively can have profound implications for reducing a spine surgeon’s workflow and increasing patient access to personal healthcare data. We demonstrate that an out-of-the-box GPT model with a base prompt can significantly simplify report text in terms of reading level, though it often omits key clinical pieces of information. We also demonstrate that providing GPT with training data within the prompt can maintain the simplicity of language while significantly improving the accuracy of translations compared to a base prompt. We further observe that even with the enhanced prompting, GPT still makes errors with regards to location of stenosis, description of prior spine surgery, and specific areas of spine pathology. We present a comprehensive study that is lumbar spine-specific, focuses on readability as well as accuracy, and includes both quantitative and qualitative analysis.

Several prior studies have assessed the ability of LLMs to simplify radiology reports in a variety of domains. Lyu et al. analyzed GPT translations of 138 radiology reports from chest CT scans and brain MRIs and demonstrated that GPT was able to significantly decrease word count, though no quantification of reading level was provided [2]. Li et al. analyzed 400 imaging reports randomly sampled across ultrasound, CT, MRI, and X-ray and found that GPT was able to significantly decrease both word count as well as Flesch-Kincaid reading level across reports [13]. Salam et al. specifically analyzed cardiac MRI imaging and found that GPT was able to produce a decreased Automated Readability Index score [14]. Similarly, Doshi et al. analyzed reading level across 750 report translations (38 of which were spine MRIs) and several different LLMs and found that all models were effectively able to reduce the reading level compared to the original report text [15]. Our analysis agrees with this prior work, demonstrating that while word count remains stable, reading level is significantly decreased with LLM translations.

Analysis of report accuracy is less commonly explored in the literature. In a proof-of-concept study, Jeblick et al. used GPT 3.5 to translate fictitious MRI reports [16]. Radiologist review of the 45 translations noted incorrect text in 51% and omission of relevant information in 22%. In addition to their readability analysis, Lyu et al. also attempted to quantify accuracy [2]. They found that for brain MRIs, 9% of reports had inaccurate information while 5% had omitted relevant information. Kuckelman et al. examined 60 musculoskeletal radiology reports (20 of which were lumbar MRIs) with three reviewers rating accuracy (from 1–3, with 3 being the highest accuracy) 2.58. 2.71, and 2.77 and completeness 2.87, 2.73, and 2.87 [5]. Salam et al. in analyzing cardiac MRIs found that reviewers “strongly agreed” in the completeness of reports 81% of the time and the factual accuracy of the reports 94% of the time [14]. Similar to Salam et al., we found that in lumbar spine MRIs, GPT tends to omit information more often than it includes incorrect information. This could be due to the lack of HIPAA protected medical data included in GPT’s original Internet-derived training set. However, the heterogeneity in results across these studies indicates that LLM performance may also be significantly influenced by the modality, pathology, anatomic region, and institution of reports. More spine-specific studies need to be conducted to validate our findings.

A more novel aspect of our study is our use of examples to enhance prompting and translations. While Lyu et al. explored the impact of altering the language within an LLM prompt to alter performance, no studies analyzing radiology report translations explicitly provided GPT with domain-specific training data [2]. Our results demonstrate that simply highlighting effective LLM outputs in a chain-of-thought paradigm can be an effective way of significantly improving downstream outputs and may allow LLMs to quickly adapt to the domain they are being deployed within over time. Despite this optimization, the fact that the LLM still made several mistakes indicates the need for further domain-specific training and validation before these models can effectively automate this spine surgeon’s task.

A potential future extension of this work would be in assessing patient, rather than provider, perspectives towards report translations. Prior work by Schmidt et al. used LLMs to produce knee MRI reports of varying complexity and presented the results to a prospective set of 20 patients [17]. While patients agreed that the reports were easy to understand and reported better comprehension of findings on the simpler reports versus the more complex, participants rated the reports as “neural” when compared to a healthcare professional explaining the results. Additionally, the ability of GPT to accurately translate reports into non-English languages could also be assessed. Gulati et al. demonstrated that, while reports were most accurately translated in English relative to other languages, GPT performed better in Spanish versus Russian or Hindi [18]. Both of these future directions would more directly involve the patient end-user in the study, and better understand if and how these technologies can be implemented in an outpatient spine clinic setting. More explicit fine-tuning on large volumes of spine radiology reports could also be considered and compared to this paper’s approach of basic context expansion.

Several limitations exist within our study. For one, we utilize imaging reports from a single institution, for a single modality and anatomic region, and for patients with a common preoperative diagnosis. While this homogeneity allowed for a more uniform performance comparison, our findings are not generalizable to other spine pathologies, imaging sequences, or institutional protocols. In addition, wording of radiology reports may also be homogenous given that all studies were read at the same institution and radiology group. We also note that we only utilized a single reviewer in our study, which could impact the reliability of our classifications of omissions and inaccuracies. This lack of interrater reliability should be more robustly assessed in subsequent studies with larger samples of reports. We also only assessed GPT-4 – other LLMs such as Google’s Gemini or Meta’s Llama may provide different performance.

Overall, this study provides the first comprehensive examination of the potential for GPT to automate radiology report simplification for spine surgeons. We demonstrate that GPT, especially when provided with training data, can competently simplify reports with a relatively low error rate. Further work is required to build upon this preliminary exploration and bring LLM technologies to the bedside.

## Data Availability

Data is not publicly available, but can be obtained upon reasonable request by contacting the corresponding author.
